# Study on Quality Standards and Hepatoprotective Effect of *Curcuma phaeocaulis* Radix

**DOI:** 10.1155/2021/6617009

**Published:** 2021-02-22

**Authors:** Zhimin Chen, Yujiao Liao, Mingyue Ao, Ying Peng, Zhuo Yang, Changjiang Hu, Lingying Yu

**Affiliations:** ^1^Chengdu University of Traditional Chinese Medicine, Chengdu, China; ^2^Chengdu Women's & Children's Central Hospital, Chengdu, China

## Abstract

*Curcuma phaeocaulis* Radix (Lüsiyujin) is a mainstream variety of Curcumae Radix cultivated in Sichuan for more than 900 years, but its broader utility is limited by a lack of quality control and pharmacodynamic research. We used the Chinese Pharmacopoeia, 2015 edition, to guide the determination of germacrone and furanodienone content in extracts. We established an animal model of Qi stagnation and blood stasis in a clinical context. Pathological changes in liver function indexes were evaluated to investigate the hepatoprotective effect of Lüsiyujin. In 20 extraction batches of Lüsiyujin, ethanol extracts yielded 9.22%–15.01%, average 12.03%. The germacrone content was 0.003%–0.011% (average 0.0069%), and the furanodienone content was 0.090%–0.478% (average 0.201%). Compared with the control group, the model rats exhibited functional liver damage. Serum markers of liver function varied after treatment with Lüsiyujin, but significant effects were observed with alanine aminotransferase and total bile acid. This study demonstrates a simple method of quality control for Lüsiyujin. The study also demonstrated that Lüsiyujin inhibits hepatocyte damage and regulates the excretion and secretion of hepatic bile. Our findings provide a theoretical basis for the formulation of quality standards, clinical application, and resource development for the utilisation of Lüsiyujin.

## 1. Introduction

Curcumae Radix (Yujin) is derived from the dried Radix of *Curcuma wenyujin* (Wenyujin), *Curcuma longa* (Huangsiyujin), *Curcuma kwangsiensis* (Guiyujin), or *Curcuma phaeocaulis* (Lüsiyujin). Yujin has long been used to treat Qi stagnation due to liver depression (LDQS) in traditional Chinese medicine (TCM) [1].

**Figure 1:**
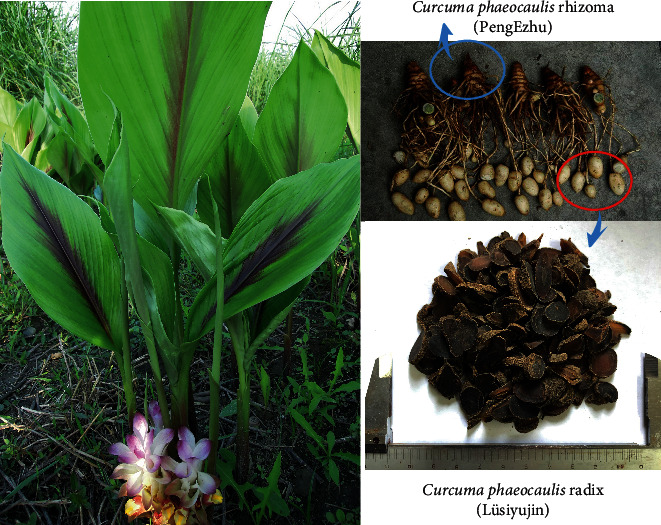
*Curcuma phaeocaulis* Val.

*Curcuma phaeocaulis* Radix (Lüsiyujin, LSYJ) is a common TCM that has been cultivated for more than 900 years in Sichuan [2]. Its original plant and medicinal parts are shown in Figure 1. Most current research has focused on Huangsiyujin [3, 4], Wenyujin [5], and Guiyujin [6] or *Curcuma phaeocaulis* rhizome [7, 8], but there have been few studies on Lüsiyujin.

Quality standards provided in the Chinese Pharmacopoeia, 2015 edition, only contain quality control items such as characteristics, identification, and physical characteristics (moisture, total ash). The standards do not yet define methods for extraction and content determination, thus preventing effective quality control. There is also a significant lack of pharmacodynamics research.

This study aimed to develop a standardised method for establishing quality control and guiding clinical use of Lüsiyujin. Germacrone and furanodienone content in the Lüsiyujin extract was determined by HPLC. We also evaluated the effect of Lüsiyujin on liver function to provide a reference for clinical application.

## 2. Materials and Methods

### 2.1. Chemicals and Reagents

The germacrone reference standard was purchased from the National Institute for Food and Drug Control (Beijing, China). The furanodienone reference standard was purchased from the Chengdu Chroma-Biotechnology (Chengdu, China). Methanol and acetonitrile (Fisher, USA) were of HPLC grade. Other reagents were of analytical purity. Water was glass-distilled and filtered through a Milli-Q water purification system (Millipore, Bedford, MA) before use. Aspirin enteric-coated tablets were purchased from Yunnan Baiyao Group (Yunnan, China). Xuefu Zhuyu Pian (Lot no. 170409) was purchased from Weifang Zhongshi Pharmaceutical (Shandong, China).

### 2.2. Plant Material

Twenty batches of Lüsiyujin were obtained from Shuangliu, Chongzhou, Wenjiang, and other producing areas of Sichuan. These samples were authenticated by Professor Ming Li (Chengdu University of TCM, Chengdu, China). Information regarding the sample materials is shown in Table 1.

### 2.3. Ethanol Extracts

Ethanol extracts were prepared according to standard 2201 of the 2015 Chinese Pharmacopoeia, Volume IV. Homeopathic alcohol was used as the extraction solvent.

### 2.4. Determination of Germacrone and Furanodienone

#### 2.4.1. Sample Preparation

Lüsiyujin powder was precisely weighed (5 g) and immersed in 25 mL methanol. Additional methanol was added to make up the loss after ultrasonic extraction for 30 min. The material was filtered (0.45 *μ*m pore size) before HPLC injection.

#### 2.4.2. Standard Solution

A mixed stock solution containing reference standards was prepared by dissolving weighed samples of each compound in methanol, yielding germacrone 54.20 *μ*g·mL^−1^ and furanodienone 453.20 *μ*g·mL^−1^. Calibration curves were established by further dilution with methanol to six different concentrations measured by HPLC (10 *μ*L).

#### 2.4.3. Analysis

HPLC determinations were performed using a Shimadzu LC-2030C 3D instrument (Shimadzu Corporation, Japan), equipped with a DAD detector, auto sampler, column heater, and Shimadzu Shim-pack GIST C18 (250 mm × 4.6 mm, 5 *μ*m) column. The mobile phase consisted of A (acetonitrile) and B (water) (V/V). Optimum separation occurred at 60%A. The flow rate was 0.8 mL·min^−1^ and injection volume was 10 *μ*L. The column temperature was set at 35°C, and elution was monitored at 210 nm.

#### 2.4.4. Method Validation

The HPLC method was validated for linearity, recovery, precision, repeatability, and stability. Validity was assessed based on relative peak areas, linear regression analysis was used to prepare calibration curves, and relative standard deviation (RSD) was used to evaluate precision; repeatability; stability; and recovery.

### 2.5. Preparation of Decoction

Lüsiyujin decoction was prepared according to the Standard for Management of a TCM Decocting Room in Medical Institutions and Technical Requirements for Quality Control and Standard Formulation of TCM Granules. Lüsiyujin was boiled for 30 min after soaking 30 min with a 9-fold volume of water and then filtered. The dregs were boiled twice for 30 min with a 7-fold volume of water and then filtered and mixed with the previous filtrate. The mixed decoction was concentrated by vacuum concentration (*T* ≤ 50°C). The rats were dosed at 0.9 g·kg^−1^, a conversion of the human clinical dosage (dose = 10 g/70 kg × 6.3 = 0.9 g/kg).

Xuefu Zhuyu suspension was prepared by grinding Xuefu Zhuyu Pian into powder and dissolving in water. The rats were dosed at 0.432 g·kg^−1^, a conversion of the human clinical dosage.

### 2.6. Animal Model Establishment

Specific pathogen-free grade Sprague-Dawley rats (200 ± 20 g) were obtained from Chengdu Dasuo Experimental Animal Co., Ltd. (licence key: SCXK(川) 2015–030, Chengdu, China). Animals were housed in polypropylene cages and left for 5 days to acclimate to the animal room (Chengdu University of TCM, Chengdu, China), maintained under controlled conditions (12 h light-dark cycle at 22 ± 2°C) on a standard pellet diet and water ad libitum. Animal experiments were approved by the Committee of Scientific Research and the Committee of Animal Care of the Chengdu University of TCM (Chengdu, China). Animals were randomly and equally divided into four groups (male and female in equal proportion): control group (CG), model group (MG), Lüsiyujin group (LG), and positive group (PoG).

CG and MG were given normal saline intragastrically. LG was given Lüsiyujin decoction intragastrically (dose = 0.9 g·kg^−1^). PoG was given Xuefu Zhuyu suspension intragastrically (dose = 0.432 g·kg^−1^). Except for the CG, LDQS blood stasis was induced in the remaining groups as described elsewhere [9]. Half an hour after drug treatment, tail clamp stimulation was applied, 4 min at a time, once every 2 h, four times a day for a week. In the second week, tail stimulation was maintained for 5 min each time, every 1 h, eight times a day. After the fourth tail stimulation, 0.6 mg/kg epinephrine hydrochloride (epinephrine hydrochloride and normal saline 1 : 3) was injected subcutaneously and repeated after 4 h, every day for a week. The CG animals were injected with physiological saline and given the same water volume as the drug groups. When the model was finished, the animals fasted for more than 12 h, with water continuing ad libitum. After administration, rats were fasted for 12 hours and anesthetized by intraperitoneal injection of 0.3 ml/100 g 2% pentobarbital sodium.

### 2.7. Histopathological Evaluation

The liver was stained with hematoxylin-eosin staining (H&E). Gross lesions were observed by BA400 Digital microscopy (Motic China Group, Xiamen, China) at 40× magnification. Images of specific lesions were captured at 100× and 400× magnifications.

### 2.8. Liver Function Tests

We evaluated several markers of liver function to understand the impact of different Lüsiyujin processing methods. Serum levels of albumin (ALB), alkaline phosphatase (ALP), alanine aminotransferase (ALT), aspartate aminotransferase (AST), *γ*-glutamyl transpeptidase (*γ*-GT), direct bilirubin (DBiL), total bilirubin (TBiL), and total bile acid (TBA) were evaluated by Chemray 240 Automatic Biochemical Analyser (Rayto Life and Analytical Sciences, China) [10, 11]. ALB, DBil and TBil were assayed by the endpoint method. ALP, ALT, AST, *γ*-GT, and TBA were assayed by the performance rate method.

### 2.9. Statistical Analysis

All data were analysed with SPSS 19.0 software and expressed as mean ± SD. Significance was determined by using one-way ANOVA. The Mann–Whitney test was used for rank data comparison. *P*-values <0.05 were considered significant.

## 3. Results

### 3.1. Ethanol Extracts

**Figure 2:**
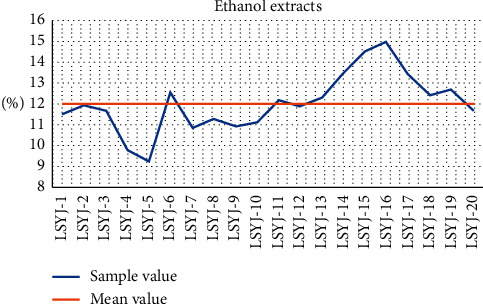
Ethanol extracts content of Lüsiyujin samples (*n* = 3).

As described in the aforementioned section, ethanol extracts of the 20 batches of Lüsiyujin yielded 9.22%–15.01% (average 12.03%). The results are shown in Figure 2.

### 3.2. Determination of Germacrone and Furanodienone

**Figure 3:**
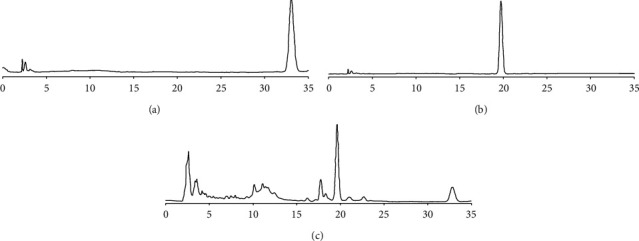
HPLC characteristics spectra of germacrone (a), furanodienone (b), and Lüsiyujin (c).

The chromatograms of mixed standards and samples are shown in Figure 3. The standards and samples had the same retention time. The degrees of separation of germacrone and furanodienone were all >1.5, and the theoretical plate number was >10000. Thus, the method provided good specificity.

As described in the aforementioned section, the calibration curves of germacrone and furanodienone exhibited good linear regressions (Table 2).

Precision was obtained by six replicate determinations of independent standard solutions, and RSD was calculated. Repeatability was determined by performing six repeat measurements of a single sample (L14). Stability studies were performed at room temperature for 0, 2, 4, 8, 12, and 24 h after preparation. Germacrone and furanodienone were stable for 24 h (RSD <3%). In the recovery test, six samples were prepared by spiking a known quantity of each standard into a Lüsiyujin sample measured and then extracted according to the sample preparation method. All data are provided in Table 3.

Samples and standards were prepared as described above and the germacrone and furanodienone content was determined by HPLC. The germacrone content in the Lüsiyujin samples was 0.003%–0.011% (average 0.0069%), and the furanodienone content was 0.090%–0.478% (average 0.201%) (Table 4).

### 3.3. Histological Analysis

**Figure 4:**
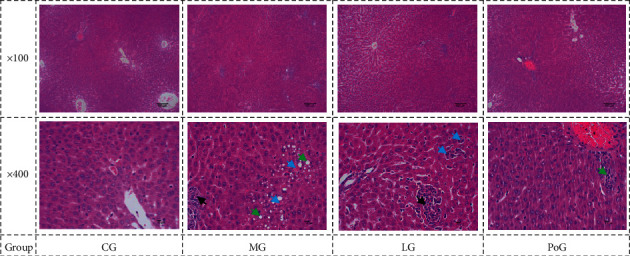
Hepatocyte pathomorphology in rat livers for various groups. Hepatocyte steatosis (green arrows); punctate necrosis (blue arrows); focal necrosis (black arrows). 400× magnification; scale bar = 10 *μ*m.

As compared with the CG of rats (Figure 4, CG), the experimental rats showed evidence of hepatic injury, including disorder of hepatic cord permutation, steatosis, round fat droplets of different sizes in the cytoplasm, punctate or special mess necrosis, and inflammation with neutrophilic and lymphocytic infiltration in the hepatic sinusoids (Figure 4, MG). Treatment with Lüsiyujin inhibited hepatic injury (Figure 4, LG). Hematoxylin and eosin (H&E) stain was used.

The liver damage was graded as follows: “−” expressed no visible cell damage, “+” slight damage, “++” moderate damage, and “+++” moderate/severe damage.  Table 5 summarises the liver damage findings in each group. Compared with the CG, the pathological changes of liver tissue in the MG were striking. After treatment with Lüsiyujin, the pathological damage was reduced, but the difference was not significant.

### 3.4. Effect of Lüsiyujin on Liver Function

**Figure 5:**
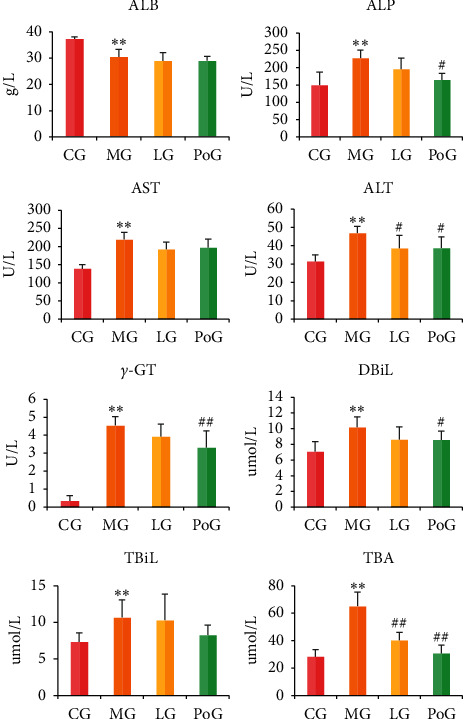
Effect of serum indexes of liver function for various groups of animals. Values were mean ± SD (*n* = 6). ^*∗*^Significantly different from the CG (^*∗∗*^*P* < 0.01); ^#^ Significantly different from the MG (^#^*P* < 0.05,^##^*P* < 0.01).

Figure 5 shows the changes in serum ALB, ALP, ALT, AST, *γ*-GT, DBiL, TBiL, and TBA in each experimental group. Compared with the CG, serum ALB levels were significantly lower in the MG rats (*P* < 0.01), and serum ALP, ALT, AST, *γ*-GT, DBiL, TBiL, and TBA were significantly increased (*P* < 0.01). These results indicate functional hepatic damage in the MG rats. After treatment with Lüsiyujin, the serum indexes of liver function showed varying degrees of change, but only ALT and TBA differed significantly.

## 4. Discussion

Traditional Chinese medicines prepared in ready-to-use forms (Zhongyaoyinpian) are necessary traditional weapons for clinical differentiation and the important raw materials of Chinese patent drugs. Therefore, the quality of Zhongyaoyinpian is related to the curative effect of TCM. An important reference for quality standards is the Chinese Pharmacopoeia (Part I) and the processing standards of TCM in various provinces. Despite its common use in Chinese herbal medicine, studies of Lüsiyujin are relatively sparse. Most quality indicators depend on germacrone, which is neither a high content component nor its exclusive component. Therefore, it is necessary to find other characteristic components of Lüsiyujin for quality control. Based on the 2015 Chinese Pharmacopoeia, relevant literature [12, 13], and preliminary investigations [14, 15], this study describes a Lüsiyujin by hot leaching with dilute ethanol. Modern research has shown that furanodienone, germacrone, and furanodiene are the principal components of *Curcuma phaeocaulis* Valeton [16]. Germacrone not only has protective effect on acute liver injury but also inhibits the proliferation and promotes apoptosis of liver cancer cells, breast cancer cells, lung cancer cells, and glioma cells [17, 18]. Chen et al. found that furanodienone, a diene-type sesquiterpene isolated from the rhizomes of Rhizoma Curcumae, exhibited a potential cytotoxic effect on temozolomide- (TMZ-) resistant GBM cells in vitro by inhibiting CSPG4 and related signalling pathways [19]. We defined a method for quality control by simultaneous determination of germacrone and furanodienone. The results showed that the separation of germacrone and furanodienone was good, and the effective control level of Lüsiyujin was comprehensively improved by multicomponent content determination.

In order to better study the pharmacodynamic effect of Lüsiyujin in the clinical practice of TCM, we established a previously described syndrome model by clamping the tail and repeatedly giving small doses of adrenaline to cause chronic anxiety in rats, who exhibited irritability, anger, and other emotions according to the theories of TCM. Biochemical serum indexes closely related to liver function were used as an indicator of liver injury [20, 21]. ALT, AST, and ALP reflect the presence and severity of hepatocyte damage [22–24]. Higher levels of ALT, AST, and ALP correlate with greater liver damage. TBiL, DBiL, and TBA are indicators of hepatobiliary excretion, secretion, and detoxification [20]. ALB is an indicator of protein synthesis and hepatocyte metabolism [25]. When liver function decreases, ALB decreases. *γ*-GT is mainly expressed in the liver and an increase reflects hyperactivity or obstruction of bile discharge [26]. Pathological examination of the liver can directly reveal the influence of liver depression and Qi stagnation. After treatment with Lüsiyujin, hepatic injury was reduced, as indicated by significant changes in ALT and TBA. We thus conclude that the main function of Lüsiyujin is to inhibit hepatocyte damage and regulate the excretion and secretion of hepatic bile, which is consistent with the TCM efficacy of Lüsiyujin in moving qi, relieving depression, disinhibiting gallbladder, and abating jaundice.

In conclusion, this study provides a basis for the quality control of Lüsiyujin and proves its effect on improving liver injury. However, the chemical composition of TCM is complex, and its functions and indications are extensive. In the follow-up study, we will use LC-MS, GC-MS, and other technologies to characterize and identify the main chemical components of Lüsiyujin, establish spectrum effect correlation combined with functional indications, and screen out the effective substance basis. Then, quantitative analysis of multicomponents with a single marker and fingerprint, combined with the pharmacodynamic component group, was used to develop a more perfect quality evaluation method system of Lüsiyujin. At the same time, we should strengthen the research on the pharmacokinetics and pharmacological mechanism of the main compounds (such as germacrone and furanodienone) of Lüsiyujin and tap their clinical application potential.

## 5. Conclusions

This study improved the quality standard of Lüsiyujin by proving the described method is simple, accurate, reliable, and reproducible. It can be used as a quality control method for other preparations. Pharmacodynamic studies showed that the Lüsiyujin inhibits hepatocyte injury and regulates hepatic bile excretion and secretion. We thus established a quality control method and preliminary pharmacodynamic data for Lüsiyujin. Further studies are needed to identify bioactive compounds and related molecular mechanisms, improve the quality standards, and provide more evidence for quality assurance and a scientific basis for clinical application.

## Figures and Tables

**Table 1:** Information about Lüsiyujin samples.

No.		Batch number/sampling time	Source	Place of purchase	
LSYJ-1		17051801	Sichuan, China	Chengdu Hehuachi Chinese Herbal Medicine Market	
LSYJ-2		17111703	Sichuan, China	Chengdu Hehuachi Chinese Herbal Medicine Market	
LSYJ-3		17111704	Sichuan, China	Chengdu Hehuachi Chinese Herbal Medicine Market	
LSYJ-4		17110305	Sichuan, China	Chengdu Hehuachi Chinese Herbal Medicine Market	
LSYJ-5		171117	Sichuan, China	Chengdu Hehuachi Chinese Herbal Medicine Market	
LSYJ-6		20160405	Wenjiang district, Sichuan province	Sichuan Zhongyong Pharmaceutical Co., Ltd.	
LSYJ-7		20171206	Wenjiang district, Sichuan province	Sichuan Zhongyong Pharmaceutical Co., Ltd.	
LSYJ-8		20171206	Wenjiang district, Sichuan province	Sichuan Zhongyong Pharmaceutical Co., Ltd.	
LSYJ-9		160901	Sichuan, China	Chengdu Ji'ankang Pharmaceutical Co., Ltd.	
LSYJ-10		161201	Sichuan, China	Chengdu Ji'ankang Pharmaceutical Co., Ltd.	
LSYJ-11		170401	Sichuan, China	Chengdu Ji'ankang Pharmaceutical Co., Ltd.	
LSYJ-12		XLS18031021	Sanjiang town, Chongzhou city, Sichuan province	Sichuan Neo-Green Pharmaceutical Technology Development Co., Ltd.	
LSYJ-13		XLS18031022	Sanjiang town, Chongzhou city, Sichuan province	Sichuan Neo-Green Pharmaceutical Technology Development Co., Ltd.	
LSYJ-14		XLS18031023	Sanjiang town, Chongzhou city, Sichuan province	Sichuan Neo-Green Pharmaceutical Technology Development Co., Ltd.	
LSYJ-15		XLS180310110	Jinqiao town, Shuangliu district, Sichuan province	Sichuan Neo-Green Pharmaceutical Technology Development Co., Ltd.	
LSYJ-16	XLS18031014		Jinqiao town, Shuangliu district, Sichuan province		Sichuan Neo-Green Pharmaceutical Technology Development Co., Ltd.
LSYJ-17	XLS18031019		Jinqiao town, Shuangliu district, Sichuan province		Sichuan Neo-Green Pharmaceutical Technology Development Co., Ltd.
LSYJ-18	XLS18031033		Huaqiao town, Xinjin county, Sichuan province		Sichuan Neo-Green Pharmaceutical Technology Development Co., Ltd.
LSYJ-19	XLS18031032		Huaqiao town, Xinjin county, Sichuan province		Sichuan Neo-Green Pharmaceutical Technology Development Co., Ltd.
LSYJ-20	XLS18031031		Huaqiao town, Xinjin county, Sichuan province		Sichuan Neo-Green Pharmaceutical Technology Development Co., Ltd.

**Table 2:** Linear ranges survey of germacrone and furanodienone in Lüsiyujin.

Compound	Regression equation	Linear range (ng)	Correlation coefficient (*r*^2^)
Germacrone	*y* = 4059953.94*x* + 55553.38	33.88∼542	0.9997
Furanodienon	*y* = 426065*x* + 12938	283.25∼4532	0.9993

**Table 3:** Precision, repeatability, stability, and recovery of germacrone and furanodienone.

Compound	Precision	Repeatability	Stability	Recovery	
	RSD (%)	RSD (%)	RSD (%)	Mean (%)	RSD (%)
Germacrone	0.18	1.44	0.36	105.90	1.20
Furanodienone	0.30	0.71	0.52	99.70	1.31

**Table 4:** The content of germacrone and furanodienone in Lüsiyujin samples (*n* = 3).

No.	Germacrone (%)	Furanodienone (%)
LSYJ-1	0.004	0.099
LSYJ-2	0.003	0.109
LSYJ-3	0.003	0.090
LSYJ-4	0.008	0.169
LSYJ-5	0.007	0.132
LSYJ-6	0.010	0.194
LSYJ-7	0.005	0.114
LSYJ-8	0.008	0.203
LSYJ-9	0.007	0.174
LSYJ-10	0.007	0.135
LSYJ-11	0.007	0.134
LSYJ-12	0.007	0.272
LSYJ-13	0.009	0.219
LSYJ-14	0.009	0.316
LSYJ-15	0.010	0.377
LSYJ-16	0.007	0.255
LSYJ-17	0.005	0.184
LSYJ-18	0.007	0.224
LSYJ-19	0.004	0.142
LSYJ-20	0.011	0.478

**Table 5:** Effect of Lüsiyujin on pathological damage of liver tissue (*n* = 6).

Group	Degree of liver damage				*P* value
	−	+	++	+++	
CG	6	0	0	0	0.002^##^
MG	0	2	4	0	1.000
LG	2	3	1	0	0.075
PoG	3	3	0	0	0.011^#^

## Data Availability

Some or all data are available from the first and corresponding author by request.
